# Toxicological impacts of environmentally equivalent microplastics and cadmium co-exposure in tropical freshwater crab *Sartoriana spinigera*

**DOI:** 10.3389/ftox.2026.1804866

**Published:** 2026-05-28

**Authors:** Ananya Chakraborty, Upayan Anam, Tanmoy Gupta, Md. Khalid Saifullah, Gourab Chowdhury, Md. Selim Reza, Mohammad Moniruzzaman, Petra Schneider, Mohammed Mahbub Iqbal, Mohammad Amzad Hossain

**Affiliations:** 1 Laboratory of Aquatic Biodiversity and Ecophysiology, Department of Fish Biology and Genetics, Sylhet Agricultural University, Sylhet, Bangladesh; 2 Department of Aquatic Resource Management, Sylhet Agricultural University, Sylhet, Bangladesh; 3 Centre for Bioinnovation, University of the Sunshine Coast, Sunshine Coast, QLD, Australia; 4 Department of Biochemistry and Molecular Biology, Jahangirnagar University, Savar, Bangladesh; 5 BCSIR Laboratories Dhaka, Bangladesh Council of Scientific and Industrial Research (BCSIR), Dhaka, Bangladesh; 6 Central Analytical Research Facilities (CARF), Bangladesh Council of Scientific Research and Industrial Research (BCSIR), Dhaka, Bangladesh; 7 Department for Water, Environment, Civil Engineering and Safety, University of Applied Sciences Magdeburg-Stendal, Magdeburg, Germany

**Keywords:** bioaccumulation, brix, haematobiochemical indices, histomorphology, *Sartoriana spinigera*

## Abstract

The toxic effects of microplastics (MPs) and trace metals on freshwater crustaceans remain poorly understood; therefore, current research examined the individual and combined effects of MPs, specifically polystyrene (PS) and polyethylene terephthalate (PET), and cadmium (Cd) on female freshwater crabs, *Sartoriana spinigera,* emphasizing behaviour, growth, haematobiochemical parameters, and tissue morphology. Crabs were exposed to five treatments (PS, PET, Cd, PS + Cd, PET + Cd) and a control for 14 days. The exposure caused notable behavioural and growth alterations, with the lowest survival rate (33.33% ± 6.67%) observed in the PS group. Bioaccumulation was highest in the gills for PS (44.67 ± 4.49 items/g) and in the hepatopancreas for PET (36.33 ± 2.41 items/g). Liver health markers showed increased alanine aminotransferase in the Cd group (9.33 ± 0.37 U/L), and elevated aspartate aminotransferase levels were noted in the PS group (25.83 ± 0.44 U/L). Additional stress markers, including total cholesterol, triglycerides, brix, glucose, and total protein, confirmed physiological disturbance in toxicant-exposed groups. Histomorphological analysis revealed structural changes in the hepatopancreas and gills, indicating impaired organ function. Overall, co-exposure caused greater physiological stress than individual treatment alone, demonstrating a combined toxic effect. These findings enhance our understanding of the complex interactions between MPs and toxic metals in tropical freshwater ecosystems, providing valuable insights for future research into multi-stressor impacts on crustacean physiology and toxicology.

## 1 Introduction

The global plastic production reached approximately 400 million tons, and prediction goes that if current production and waste management trends continue, plastic waste could exceed nearly 1200 million tons by 2050 (Badejo et al., 2024). Plastics that enter natural ecosystems break down into smaller particles, such as microplastics (MPs, particles less than 5 mm), through physical, chemical, and biological processes (Lalrinfela et al., 2024). Their small size and large surface area enable MPs to disperse easily in water, increasing the likelihood of being ingested by aquatic life and amplifying the risks they pose to aquatic ecosystems (Jahan et al., 2024a). Previous evidence shows the toxic impacts of MPs on marine and freshwater organisms, with implications for ecosystem health (Gratt et al., 2025). By affecting multiple trophic levels, MPs disrupt ecosystems, contaminate food chains, and may also pose risks to human health (Khornchatri et al., 2020).

Riverine ecosystems are vital for maintaining global ecological and socioeconomic stability, as they support diverse biodiversity and enhance ecological resilience (Akther KR. et al., 2024; Saifullah et al., 2025). Recent research indicates that urban rivers have significantly higher pollution levels, with elevated concentrations of MPs and trace metals (Anam et al., 2025). These findings identify contamination sources and their harmful impacts on aquatic life, ecosystems, habitats, communities relying on these waters, and the ecological balance of interconnected freshwater and marine systems (Khan et al., 2023). Since urban rivers are situated near major sources of pollution, they often build up higher levels of MPs and trace metals, making their aquatic ecosystems more susceptible to ecological threats.

Hildebrandt et al. (2021) evidenced that MPs pose threats to both aquatic and human life and can act as Trojan horses for dissolved metals. In recent years, growing concern has focused on MP pollution in the marine environment (Thilagam et al., 2024). While the impacts of MPs on marine species are relatively well-documented, their effects on freshwater organisms remain comparatively underexplored (Ockenden et al., 2021). Although many studies have explored the interaction between MPs and trace metals, most have focused on marine species or a narrow range of freshwater taxa (Bhaumik and Chakraborty, 2024). Data on benthic invertebrates, such as freshwater crabs, are particularly limited under co-exposure scenarios that reflect pollutant mixtures from specific freshwater habitats (Silva et al., 2022). In aquatic environments, trace metal contamination poses a major global threat because it can bioaccumulate through the food web, ultimately endangering human health (Jolly et al., 2023). Cadmium (Cd) is one of the most prevalent and highly toxic metals in aquatic ecosystems and is well known to have teratogenic, mutagenic, and carcinogenic effects (Cheng et al., 2023). It poses a significant threat due to its ability to bioaccumulate steadily in living organisms, leading to potential risks to both humans and the environment (Abdullah et al., 2022). Chronic Cd exposure can also cause harm to aquatic life by disrupting growth, survival, and other physiological functions (Liu et al., 2022). Moreover, when MPs interact with such trace metals, they may induce co-exposure effects on organisms, influencing the environmental behaviour and toxicity of these toxicants (Sendra et al., 2021; Mahi et al., 2022).

Large-scale emissions of MPs, especially polystyrene (PS) and polyethylene terephthalate (PET), tend to settle to the bottom sediments, making them readily accessible to detritivorous organisms (Issac and Kandasubramanian, 2021). Among the plastic polymers, PS is often considered one of the most hazardous due to its chemical composition (Yang et al., 2022). PS is a polymer formed through the polymerization of styrene monomers. It is widely used to make products such as styrofoam, toys, compact discs, and cup lids, which significantly contribute to MP pollution in freshwater environments (Kik et al., 2020). PET, extensively used in textile manufacturing and industrial packaging, is another major source of MPs in freshwater ecosystems (Dhaka et al., 2022). Toxic trace metals, along with MPs, harm aquatic life by altering physicochemical parameters, inducing oxidative stress, inhibiting enzymes, and disrupting physiological and biochemical functions (Sharma et al., 2025). However, the combined accumulation of MPs and toxic metals can amplify ecological risks and induce elevated toxic effects on aquatic organisms (Devi et al., 2025). This study assessed the short-term effects of combined PS and PET MPs and Cd exposure. MPs carry toxic metals such as Cd, increasing their bioavailability and accumulation in crab tissues, thereby worsening health, growth, and survival (Haque et al., 2023).

MPs have been widely detected in crustaceans, including wild crabs, highlighting widespread contamination of aquatic food sources and raising public health concerns (Chen H. et al., 2025; Tang, 2025). Exposure to MPs has been demonstrated to negatively impact crab growth, physiological processes, and reproductive success (Fu et al., 2024). Additionally, crabs’ benthic life makes them highly vulnerable to metal contaminants, especially Cd (Bao et al., 2021). The freshwater crab *Sartoriana spinigera* is one of the most prevalent crustaceans in Bangladesh’s freshwater ecosystems (Akther S. et al., 2024). Ecologically, *S. spinigera* maintains ecosystem balance and aids nutrient cycling in tropical and subtropical freshwater habitats (Che et al., 2024). Beyond its ecological importance, this species is valued as a nutritious food source for its high protein and lipid content (Pati et al., 2012). Its benthic and detritivorous feeding makes *S. spinigera* susceptible to sediment pollutants, enabling bioaccumulation of MPs and trace metals, making it a promising bioindicator of environmental contamination (Yang et al., 2022).

Given the ecological importance and sensitivity to pollutants of *S. spinigera*, understanding its response to realistic contamination scenarios is crucial for effective management and conservation. Freshwater river ecosystems face severe pollution during dry seasons, as water levels drop, increasing MP concentrations and toxic metal levels, and stressing aquatic life (Abdullah et al., 2022). Research on the effects of high contaminant levels on freshwater crustaceans is limited, with most studies focusing on small-scale surveys in Bangladesh, leaving a gap in understanding their toxicity. This study fills that gap by examining the impact of environmentally relevant pollutant doses on female *S. spinigera* in a controlled laboratory setting. Pollution levels mimicked urban rivers to evaluate effects on behavior, physiology, and organ health using a multi-biomarker approach. This included growth, behavior, blood biochemistry (ALT, AST, TG, TP, TC, glucose, brix), histomorphology, and pollutant bioaccumulation. Indicators reflect an organism’s health, metabolism, and stress, which are vital for assessing population resilience. This study provides the first comprehensive toxicological profile of combined pollutants, essential for ecological risk assessments in tropical freshwater ecosystems impacted by urban and agricultural runoff.

## 2 Materials and methods

### 2.1 Experimental crab collection and acclimatization

Adult hard-shelled inter-moult female *S. spinigera* were collected (*n* = 90, wet weight 14.42 ± 1.11 g) from Hakaluki Haor (Coordinates: 24°41′43″N 92°00′47″E), a vast aquatic reservoir in the northeastern part of Bangladesh. The crab collection used traditional bamboo traps set by local fishermen for 6–8 h in a protected, less-polluted fish sanctuary. Females were chosen for their lower cannibalism, ensuring better survival during acclimation (Zhang et al., 2018). Post-capture, the crabs were quickly placed in aerated containers with water from the capture site and transported within an hour to the wet laboratory (Department of Fish Biology and Genetics) at Sylhet Agricultural University, Sylhet 3,100, Bangladesh.

Upon arrival, the crabs (*n* = 90) were acclimated for 2 weeks in glass aquaria (75 cm × 35 cm × 30 cm) with five crabs per tank, all in carbon-filtered tap water. This period stabilized their physiology and aided in the depuration of toxicants (Türkmen et al., 2024). These aquaria had proper aeration (round-shaped; 4 aquarium air-bubble stones per tank), and the lab was well-ventilated with ample natural light from multiple windows. Water quality was maintained at optimal levels by partially siphoning water every 24 h and fully replacing it every 48 h to control ammonia (NH_3_) levels. During this time, the crabs received daily feedings of minced tilapia muscle, locally sourced and formed into small balls, at a rate of 3% of their body weight (Yang et al., 2022). Uneaten feed was promptly removed after feeding to ensure water quality. The pre-experimental feed had minimal MPs (0.6 ± 0.55 items/g), mostly fibers, carefully removed under a stereomicroscope. Cadmium levels were low (0.067 ± 0.006 μg/kg), reducing interference with experiments.

### 2.2 Experimental design, toxicants exposure preparation, and crab allocation

For the exposure trial, analytical-grade Cadmium Chloride (CdCl_2_) (Merck, Germany) was used. Packaged fine-powdered commercially labelled MPs polymers - PS (150 mesh; irregular fragments, size: 120.23 ± 42.84 µm) and PET (150 mesh; irregular fragments, size: 62.91 ± 17.65 µm) - were procured from Fengtai Polymer Materials Co., Ltd., China (Catalogue Number- 13412040938). To further validate the identity of the procured materials, FTIR analysis was performed, confirming their composition as PS and PET (Supplementary Figure S1). The concentrations were 200 μg/L for both PS and PET, and 62.5 μg/L for Cd, representing environmentally relevant levels for this study. Six groups were established: Control (0 MPs, 0 Cd), PS (200 μg/L), PET (200 μg/L), Cd (62.5 μg/L), PS + Cd (200 μg/L + 62.5 μg/L), and PET + Cd (200 μg/L + 62.5 μg/L) (Supplementary Table S1). All preparations were performed under sterile conditions to prevent cross-contamination and ensure experimental consistency. The stock solutions of combined MPs (*i.e.,* PS, PET) and Cd were prepared 48 h before use in the trial (Yang et al., 2022). The preparation was done in a 25 °C shaker-incubator with a 12-h light/dark cycle. Stock solutions for each toxicant were prepared in filtered distilled water and added to the aquaria.

The current experiment used PS, PET, and Cd as typical urban freshwater pollutants in Bangladesh, where trace metal and MP levels increase during the dry season due to less rainfall and water flow (Abdullah et al., 2022). Past studies reported Cd levels from 45 to 124 μg/L in urban areas of Bangladesh (Pandit et al., 2024; Preonty et al., 2025). A concentration of 62.5 μg/L was chosen for Cd, reflecting an environmentally relevant, yet elevated level. On the other hand, recent studies have documented moderate MP concentrations in urban river systems, including canals in Dhaka, Bangladesh (∼40 particles/L) (Parvin et al., 2022), the Balu River (∼59 particles/L) (Odora et al., 2024), and the Buriganga River (∼53 particles/L) (Mahmud et al., 2025). Although polymer-specific data for Bangladesh is unavailable, MPs were introduced at 200 µg of PS and PET, about 48–56 particles, aligning with environmental reports. The 14-day simulation reflected peak contamination and natural conditions.

Acclimatised (n = 90) female *S. spinigera* were randomly subjected to six experimental groups (control, PS, PET, Cd, PS + Cd, PET + Cd), each with three independent replicate tanks per treatment (*n* = 3 tanks/treatment) over a 14-day experimental period. Therefore, each tank initially contained five crabs, resulting in 15 crabs per treatment and 90 crabs overall (Supplementary Table S1). To maintain consistent exposure levels, all aquarium solutions were fully renewed every 24 h (Yang et al., 2022). Feeding protocols matched acclimatization with natural light, ventilation, and continuous aeration for oxygen. Aquaria surfaces covered with black sheets up to 20 cm to mimic habitat, reduce stress, and promote natural behaviours. Water quality details are in Supplementary Table S2.

### 2.3 Observation of behavioural abnormalities

Daily observations were conducted to track behavioural abnormalities in female *S. spinigera* during the exposure period, focusing on changes in locomotion, signs of illness, or other significant alterations. The monitoring process was developed by Deyashi et al. (2019) and modified to match the species-specific behavioural characteristics of female *S. spinigera* under laboratory conditions. The observed behaviours were selected based on previous research, as detailed in Supplementary Table S3. Key behavioural responses-such as aggregation, aggression, attraction to food, fecal excretion, froth releasing, hiding behind the aeration stones, locomotion, and mouthpart activity-were recorded once daily at 9:00 a.m., both before and after feeding, each session lasting 60 min (Deyashi et al., 2019; Haye and Ojeda, 1998). To facilitate comparison and interpretation, the recorded responses were expressed as percentages, and the data were aggregated and presented at 7-day and 14-day intervals.

### 2.4 Sampling, haemolymph collection and tools for growth matrix

Crabs fasted for 24 h prior to sampling and haemolymph collection. Their final weight was measured with an electronic balance (CAMRY digital electric balance, Model EK 3052, Bangladesh) before collecting haemolymph from each experimental group (*n* = 3). To anesthetize the crabs, they were cooled by placing them in ice slurry for approximately 25 min as described by Chen L. et al. (2025). Three crabs per replicate (*n* = 3) were randomly sampled for haemolymph from abdominal segments between their walking legs using a 26-gauge syringe (1 mL). The haemolymph was then diluted 1:1 with pre-cooled freshwater anticoagulant (140 mM NaCl, 10 mM KCl, 10 mM HEPES, 10 mM EDTA-disodium, 30 mM trisodium citrate, pH 7.3) for haematological analysis (Black et al., 2024). Approximately 1.5 mL of haemolymph was drawn from each crab. Additionally, crabs from each replicate were sacrificed to collect gills and hepatopancreas for assessment of MP abundance (*n* = 3). To ensure sufficient replication for Cd concentration analysis on a dry weight basis, whole crabs from each treatment were collected (*n* = 3), as described in Section 2.5. All samples were stored at −20 °C for future analysis. Gills and hepatopancreas tissues from each treatment and control group (*n* = 5) were fixed in 10% neutral buffered formalin (NBF) for histomorphological examination. The specific growth rate (SGR) and hepatopancreatic index (HPI) were calculated as described in the literature (Aaqillah-Amr et al., 2022; Hossain et al., 2025). As mortality occurred across treatment groups, animals that died before the experiment’s end date were excluded from biochemical assays, bioaccumulation, histomorphology, and growth and index calculations.

### 2.5 Sample digestion, extraction, analysis and quantification of MPs and Cd

MP extraction from the gills and hepatopancreas of female *S*. *spinigera* was performed using a standardized chemical digestion protocol as described by Haque et al. (2023). Gill and hepatopancreas tissue samples were homogenised separately using a mortar-pestle, and pooled triplicates (*n* = 3; 1 g wet weight per replicate) from each experimental treatment. To digest organic material, 10% potassium hydroxide (KOH) was added to each sample, followed by incubation at 40 °C for 5 days. After incubation, the digested samples were filtered using Whatman GF/C microfiber glass filter paper (pore size 1.2 μm, diameter 47 mm). MPs retained on the filters were visualized and quantified using a stereomicroscope (Primo Star 415500-0057-000, Carl Zeiss Microscopy GmbH) equipped with a digital camera (Axiocam 208 colour, Carl Zeiss Suzhou Co., Ltd.). Images were captured using ZEN 3.1 Blue Edition software. To further validate the efficiency of the MP digestion method, a spiking test was conducted, and the results are presented in Supplementary Table S4, yielding a mean recovery rate of 94.33% ± 2.53%, confirming the reliability and efficiency of the current protocol. During stereomicroscopic identification, MPs were recorded only when they exhibited similar morphology to raw polymer particles, excluding visible cellular or organic structures, displaying a homogeneous transparent coloration, and having sharp, non-biological edges. Additionally, natural fibers were excluded, and particles larger than 10 µm were counted due to the resolution limits of the stereomicroscope. Moreover, suspected particles were verified using a hot-needle test: true plastics melted or deformed upon contact, whereas non-plastic materials showed no response, providing an extra confirmation step. To ensure reliable MP quantification, each replicate filter was analysed three times independently by two trained analysts, and the average counts were used for statistical analysis. Any discrepancies were then reviewed collaboratively until a consensus was reached.

While Cd extraction from whole-body crab samples was processed according to the protocol outlined by Anam et al. (2025). Samples were oven-dried at 105 °C for 12 h, then homogenized and sieved through a <2 mm mesh. A 1-g (*n* = 3; dry weight) portion of the dried, powdered sample was digested with 10 mL of ultrapure nitric acid (69%–70%) and 2 mL of hydrogen peroxide (30%) in a 50 mL beaker. The mixture was heated on a hotplate at 200 °C–250 °C until it became clear. The resulting solution was diluted to 50 mL with distilled water, filtered through Whatman ashless filter paper, and stored at 4 °C. Cadmium concentrations were measured using an ICP-MS (NexION 2000, PerkinElmer, United States). The LOD and LOQ were 0.0052 μg/L and 0.053 μg/L, respectively, based on S/N ratios of 3 and 10. Validation with certified reference material (SRM NIST 1643f) yielded recoveries of 96.21%–102.02%, indicating high accuracy and precision. Throughout the laboratory procedures, strict contamination controls were implemented, including rinsing equipment with Milli-Q water, wearing 100% cotton fibre-made lab coats (non-synthetic), nitrile gloves (powder-free), sealing container openings with aluminium foil, using filtered reagents and distilled water by GF/C filters, and conducting procedural blank tests to monitor contamination. However, no MPs were found in the blanks, which suggests that the results were reliable. Reagents were handled with strict precautions; sample digestion was performed in a fume hood, and procedures such as sample preparation and filtration were performed in a laminar flow hood.

### 2.6 Assay of haematobiochemical parameters

Brix of crab haemolymph (*n* = 3) was estimated immediately after collection using an automatic refractometer (ATAGO® Manual MASTER-53S, f = 29.65 mm Ø13.5 mm, A-458) (Berry et al., 2019). Distilled water was used as a blank between each sample for calibration. The Brix is closely linked to haemolymph biochemistry and overall crustacean condition (Berry et al., 2019; Battison, 2018), and was used in this experiment as a rapid indicator of moulting tendency under MPs and Cd exposure. Haemolymph plasma was extracted using a refrigerated high-speed microcentrifuge (GYROZEN, Refrigerated, High-Speed Micro Centrifuge, 1730 R GYROZEN, Republic of KOREA) at 4 °C and 5,000 × g rotation for 10 min (Onwubiko et al., 2022). The plasma was stored at −20 °C for subsequent biochemical analysis. Biochemical parameters (*n* = 3), including alanine aminotransferase (ALT), aspartate aminotransferase (AST), total cholesterol (TC), triglycerides (TG), total protein (TP), and glucose, were determined using a VITROS™ Chemistry 350 System automatic biochemistry analyser (Ortho-Clinical Diagnostics™ 6802153, New Jersey, United States) with individual tests performed using VITROS® Chemistry Products analysis kits (Ortho-Clinical Diagnostics™ 6802153, New Jersey, United States).

### 2.7 Histological analysis of vital organs

Pre-fixed tissues in NBF were subjected to processing through a standard procedure for histomorphological study (Hidir et al., 2018; de Freitas Rebelo et al., 2000). Crab gill and hepatopancreas tissues were fixed in 4% paraformaldehyde, dehydrated in ethanol, cleared in xylene, embedded in paraffin, sectioned at 4 μm, and stained with haematoxylin and eosin. Five slides per tissue (*n* = 5) were examined microscopically for abnormalities, and stereomicrographs were taken at 10× and 40× magnification for detailed assessment. All hepatic cells on the hepatopancreas slides and the entire gill slides were analysed. Histopathological changes were classified as absent (<5%), weak (5%–25%), moderate (25%–50%), or severe (>50%) (Jahan et al., 2024b).

### 2.8 Data analysis and software

Raw data from different treatment groups were first organised using Microsoft Excel 365 and then analysed with IBM SPSS Statistics v27.0.1.0. Normality was checked with the Shapiro–Wilk test (*n* < 50) and visualised using QC plots in the ‘Explore’ function. For datasets that followed a normal distribution, one-way ANOVA assessed differences between groups, followed by Tukey’s HSD *post hoc* test (*p* < 0.05). Non-parametric data were analysed with the Kruskal–Walli’s test, and Dunn’s *post hoc* test was used if significant differences arose. Superscripts were assigned based on pairwise comparisons with Bonferroni correction (*p* < 0.05). Additionally, detailed statistical outputs for all analyses conducted in the experiment, including test statistics, degrees of freedom, and exact p-values, were stated in Supplementary Table S5. All graphs, including Principal Component Analysis (PCA), were created in OriginPro 2025b (64-bit) v10.2.5.212 (OriginLab, United States). Before PCA, dataset suitability was verified using Bartlett’s test of sphericity (*χ* ^2^ = 132.874, *p* < 0.001) and a KMO value of 0.789, indicating sampling adequacy and correlation among variables.

## 3 Results

### 3.1 Behavioural abnormalities

**FIGURE 1:**
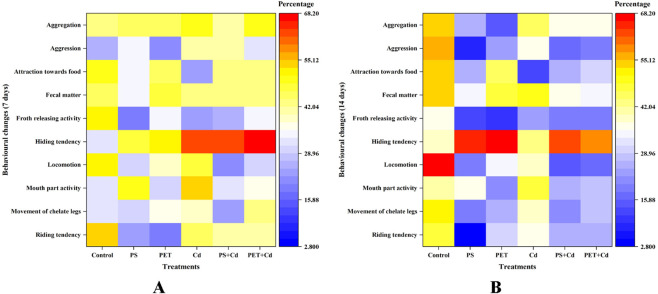
Behavioural alterations of female *S. spinigera* across treatment groups: Control, PS, PET, Cd, PS + Cd, and PET + Cd, after **(A)** 7 days and **(B)** 14 days of exposure.

In the control group, female *S. spinigera* exhibited normal behavior without abnormalities (Figure 1). After 7 and 14 days of exposure to acute toxicants, notable behavioral changes were observed, including aggregation, aggression, riding tendency, mouthpart activity, hiding (behind the aerator stone), froth release, and fecal excretion. Toxicant exposure significantly reduced locomotion, aggregation, aggression, and chelate leg movement compared to controls, suggesting physiological stress. Conversely, locomotion increased in the control group after 14 days. Hiding behavior intensified over time in individual and combined MP exposure groups, likely reflecting stress and avoidance. Food attraction remained consistent in controls but declined in all treatments, most prominently under sole Cd exposure (Supplementary Table S6).

### 3.2 Growth matrix and survival rate

The control group showed a higher SGR value, while other treatment groups had lower values, with the lowest recorded in the Cd treatment (Table 1). Notably, the control group exhibited the highest survival rate, while the lowest was recorded in the PS and PS + Cd groups, likely due to PS toxicity.

**TABLE 1:** Growth performance parameters of experimental female *S. spinigera* subjected to different treatments.

Treatment	Control	PS	PET	Cd	PS + Cd	PET + Cd
Initial weight (g)	14.39 ± 0.43	14.43 ± 0.25	14.41 ± 0.40	14.46 ± 0.37	14.41 ± 0.42	14.41 ± 0.46
Final weight (g)	15.61 ± 0.46	14.44 ± 0.22	14.83 ± 0.39	14.72 ± 0.36	14.77 ± 0.63	14.92 ± 0.64
SGR (%)	0.58 ± 0.06^b^	0.19 ± 0.04^a^	0.21 ± 0.02^a^	0.13 ± 0.05^a^	0.27 ± 0.06^ab^	0.24 ± 0.03^ab^
Hepatopancreas weight (g)	0.59 ± 0.02	0.52 ± 0.02	0.54 ± 0.02	0.58 ± 0.05	0.61 ± 0.03	0.63 ± 0.04
HPI	3.78 ± 0.10	3.60 ± 0.13	3.68 ± 0.12	3.90 ± 0.27	4.17 ± 0.26	4.27 ± 0.23
Survival rate (%)	100 ± 00^b^	33.33 ± 6.67^a^	66.67 ± 6.67^ab^	60.00 ± 00^ab^	33.33 ± 6.67^a^	46.67 ± 5.89^ab^

Values are expressed as mean ± SEM; within rows, different superscripts (a, b) indicate significant differences (*p* < 0.05). The same sample size applies to initial weight and survival rate (%) measurements across all treatment groups. Otherwise, *n* = 9 for control, PET and Cd, 5 for PS and PS + Cd, and 7 for PET + Cd in regard to final weight (g), SGR (%) (specific growth rate), hepatopancreas weight (g), and HPI (hepatopancreas index).

### 3.3 MPs and Cd bioaccumulation after exposure

**FIGURE 2:**
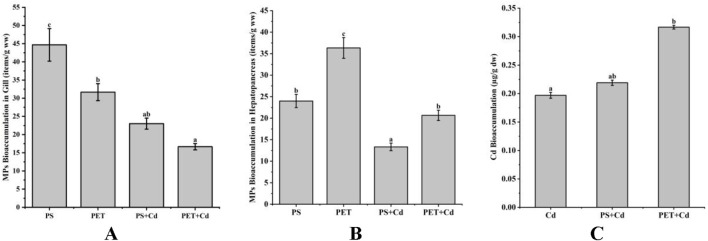
Bioaccumulation of toxicants in female *S. spinigera* after a 14-day exposure: **(A)** MPs in gills (items/g wet weight) **(B)** MPs in hepatopancreas (items/g wet weight), and **(C)** Cd in whole body (µg/g dry weight). Values are mean ± SEM (*n* = 3). Different superscript letters indicate statistically significant differences among treatments (*p* < 0.05).

The presence of MPs in the hepatopancreas and gills was confirmed in the PS, PET, PS + Cd, and PET + Cd treatment groups (Supplementary Figure S2), with substantial variation observed between these organs in female *S. spinigera*. MP accumulation in gills (wet weight) was higher in PS-associated treatments (Figure 2A), whereas accumulation in the hepatopancreas (wet weight) was greater in PET-associated treatments (Figure 2B). Additionally, Figure 2C shows Cd-associated bioaccumulation (dry weight) in female *S. spinigera* (whole body) after exposure, where Cd accumulation in the Cd treatment was significantly lower (*p* < 0.05) compared to the PET + Cd group. Cd-exposed treatments with traces of MPs showed a notable increase in Cd accumulation. In particular, PET polymers in the PET + Cd group potentially appeared to enhance Cd accumulation to a greater extent than PS particles in the PS + Cd treatment.

### 3.4 Haematobiochemical parameters

**FIGURE 3:**
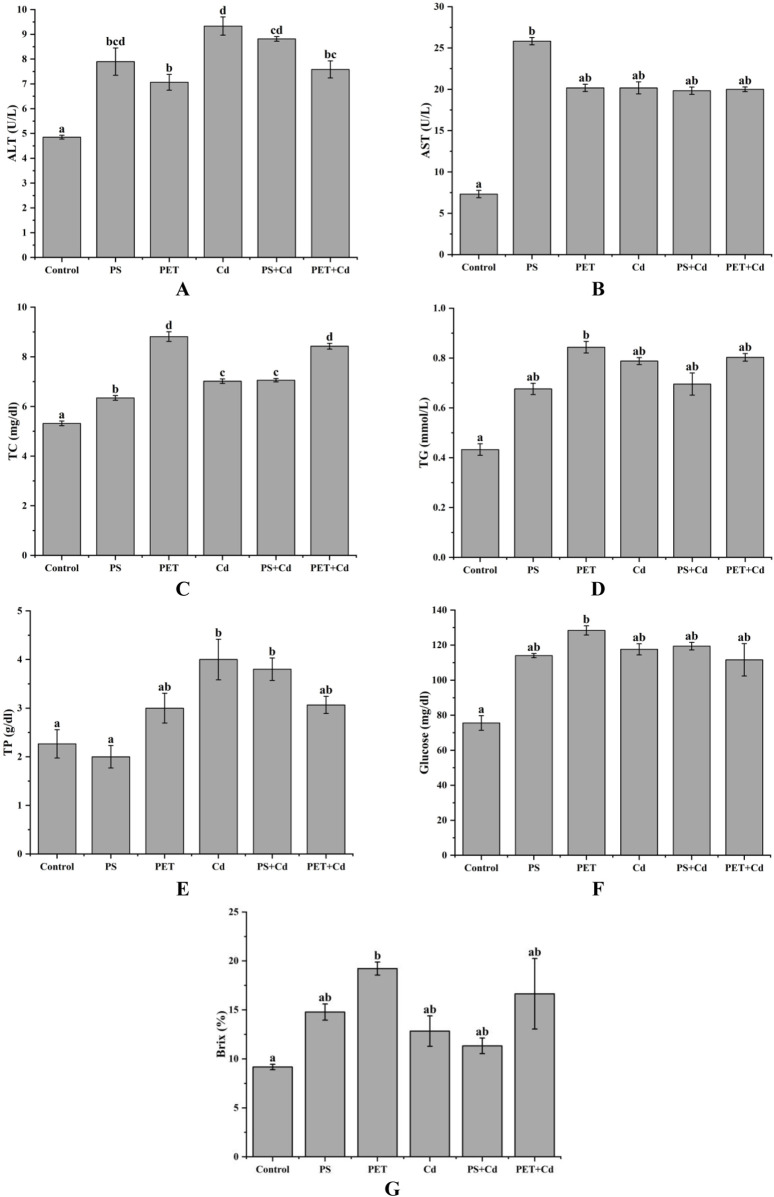
Haematobiochemical parameters of haemolymph in female *S. spinigera* **(A)** alanine aminotransferase-ALT (U/L) **(B)** aspartate aminotransferase-AST (U/L) **(C)** total cholesterol-TC (mg/dL) **(D)** triglycerides-TG (mmol/L) **(E)** total protein-TP (g/dL) **(F)** glucose (mg/dL), and **(G)** brix (%). Values are mean ± SEM (*n* = 3). Different superscript letters indicate significant differences among treatments (*p* < 0.05).

Compared to the control group, ALT, AST, TC, TG, TP, glucose, and Brix levels significantly increased across the exposure treatment groups (*p* < 0.05) (Figures 3A–G). However, TC and TG were elevated in the PET-containing groups, while the individual Cd treatment also raised TC and TG compared to other treatments. ALT was also elevated in the sole Cd treatment, while the PS treatment showed a significantly higher AST value (*p* < 0.05). Higher glucose levels indicate possible physiological stress in all exposure groups, compared to the control. TP was significantly higher in the Cd and PS + Cd (*p* < 0.05). Furthermore, the higher Brix values in PET-associated groups indicated a potential moulting response to PET exposure treatments.

### 3.5 Histopathological changes of gills and hepatopancreas

**FIGURE 4:**
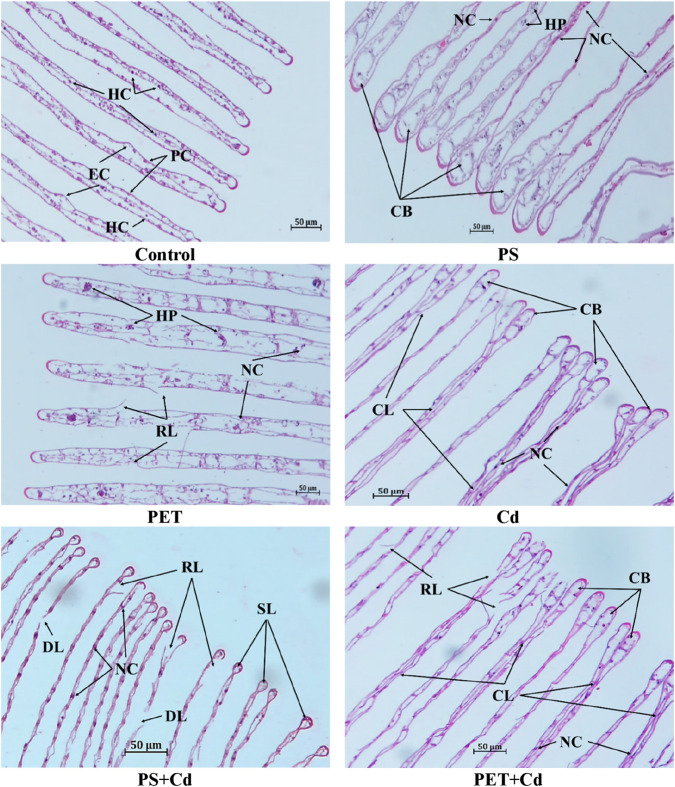
Histomorphological alterations in the gills of female *S. spinigera* exposed to individual and combined treatments of MPs (*i.e.,* PS, PET) and Cd after 14 days, compared to the control. Where marked as Hemocytes (HC); epithelial cells (EC); pillar cells (PC); clubbing (CB); connections of gill lamellae (CL); necrosis (NC); hyperplasia (HP); ruptured lamellae (RL); detached lamellae (DL); swollen lamellae (SL). Magnification: ×10; scale bars = 50 µm.

**FIGURE 5:**
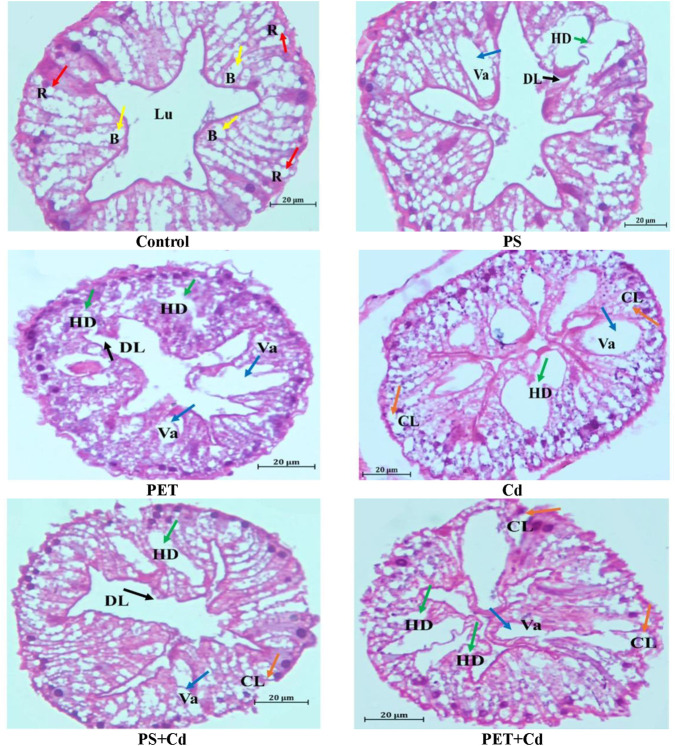
Histomorphological changes in the hepatopancreas of female *S. spinigera* exposed to individual and combined treatments of MPs (*i.e.,* PS, PET) and Cd after a 14-day trial, compared to the control. Where marked as B cells (B, yellow arrow); R cells (R, red arrow); lumen (Lu); vacuoles (Va, blue arrow); dilation of hepatic tubular lumen (DL, black arrow); cell lysis (CL, orange arrow); hepatic digestion (HD, green arrow). Magnification: ×40; scale bars = 20 µm.

In the control group, gills showed typical lamellae and cell arrangements (Figure 4). Female *S. spinigera* exposed to toxicants exhibited severe structural damage, including swollen, ruptured, and fused lamellae, detachment, hyperplasia, necrosis, and clubbing. Hepatopancreas tissues in controls were well-organized with distinct cells, intact tubules, and normal B and R cells (Figure 5). Toxicant exposure caused alterations, including lumen dilation, deformation, vacuolation, lysis, and hepatic digestion, in PS, PET, Cd, and combined treatments (Table 2). Tissue deterioration increased with dose; PS severely damaged gills, PET caused hepatic deformation, and Cd effects ranged from mild to severe (Supplementary Table S7).

**TABLE 2:** Percentage of histomorphological changes in female *S. spinigera* exposed to MPs and Cd for 14 days.

Organs	Histological abnormalities	Treatments					
		Control	PS	PET	Cd	PS + Cd	PET + Cd
Gill	Clubbing	–	+++	+	+++	++	+++
	Connection of lamellae	–	++	+	+++	++	+++
	Necrosis	–	+++	++	++	+++	++
	Hyperplasia	–	+++	+++	+	++	++
	Ruptured lamellae	–	+	+++	+	+++	++
	Detached lamellae	–	+	+	+	+++	+
	Swollen lamellae	–	++	+	+	+++	++
Hepatopancreas	Vacuoles	–	++	+++	+++	++	+++
	Dilation of hepatic tubular lumen	–	++	+++	++	+++	++
	Cell lysis	–	–	++	+++	++	+++
	Hepatic digestion	–	+	+++	++	++	+++

Abnormalities noted <5% have been referred to as absent (−), 5% – 25% as weak (+), > 25 – 50% as moderate (++), and >50% as severe (+++) in the current study.

### 3.6 Principal component analysis (PCA)

**FIGURE 6:**
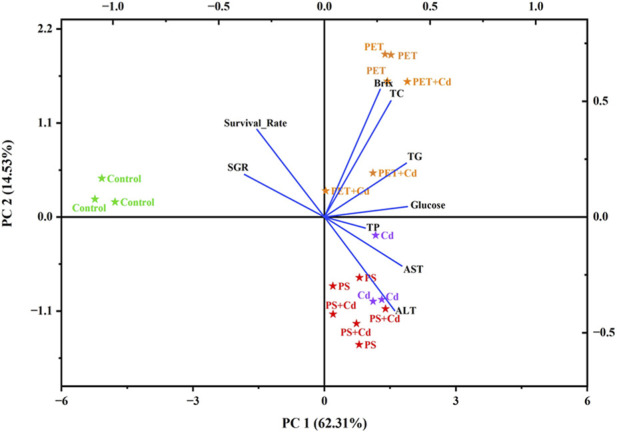
Principal Component Analysis (PCA) of growth, survival, and haematobiochemical parameters in female *S. spinigera* exposed to individual and combined exposure of MPs and Cd.

The PCA revealed that single and combined MPs (*i.e.,* PS and PET) and Cd treatments significantly affected female *S. spinigera*, explaining 76.84% of the total variance (PC1: 62.31%; PC2: 14.53%). Figure 6 shows that the control group correlated with survival and growth, indicating a healthy physiological status. Liver toxicity indicators (AST, ALT) were linked to Cd, PS, and PS + Cd clusters, suggesting significant stress and liver damage. PET and PET + Cd clusters linked to metabolic markers (Brix, TC, TG, glucose) but negatively impacted survival, indicating disrupted metabolism and energy reserves. Both PS and PET, especially with Cd, caused physiological stress, impaired growth and survival, and altered haematobiochemical profiles, with treatment-specific effects in female *S. spinigera.*

## 4 Discussion

Crabs are known as model organisms for assessing ecotoxicological impacts in aquatic environments, and they can bioconcentrate contaminants several times higher than in the surrounding environment (Ramos-Filho et al., 2025). As vital bioindicators, they reveal the bioavailability and effects of environmental contaminants. Studies show PS and PET particles are linked to toxicity, neurotoxicity, cytotoxicity, and oxidative stress in crustaceans (Multisanti et al., 2025; Khanjani et al., 2025). Similarly, Cd exposure was shown to cause toxic effects in aquatic species, including biochemical changes, deformities, and physiological dysfunction in crabs (Waqas et al., 2024; Banaee et al., 2024).

Crabs are recognized as model organisms for studying behavioural responses to pollution and environmental stress (Briffa et al., 2024). Behavioural changes reported in freshwater crabs (*Varuna litterata*) include erratic movements, altered feeding, restlessness, escape responses, impaired locomotion, and aggressiveness. Such effects have been linked to diverse stressors, including metals (Rosaria and Martin, 2010), pesticides, biopesticides (Deyashi et al., 2016), and mahua oil cake toxicity (Deyashi et al., 2019). These behavioural endpoints, such as quick responses to stressors, serve as early indicators of environmental toxicity that are not always evident in traditional measures.

Previous research shows MPs can adsorb heavy metals due to their large surface area and hydrophobic traits, acting as vectors for transport to aquatic life (Bhaumik and Chakraborty, 2024; Narwal et al., 2024). The toxic effects of hazardous metals primarily result from their bioaccumulation in tissues, leading to histopathological damage, oxidative stress, and cellular dysfunction in aquatic species (Kumar et al., 2024; Zahran et al., 2025). For instance, Cd accumulates in the hepatopancreas and induces hepatocyte damage in crustaceans (Banaee et al., 2025a). Laboratory studies have shown that crabs, such as the mudflat fiddler crab (*Minuca rapax*), may ingest MPs, mistaking them for prey (Pichardo-Casales et al., 2024). Co-exposure to MPs and trace metals has been shown to increase metal uptake in species such as the Chinese mitten crab (*Eriocheir sinensis*) and the blue crab (*Callinectes sapidus*) (Yang et al., 2022; Munuera et al., 2021). In this study, female *S. spinigera* exposed to PS alone or in combination with Cd exhibited significantly higher MP accumulation in the gills, likely due to the gills’ fine structure, which facilitates particle retention (Aguirre-Sanchez et al., 2024). Cd primarily enters through the gills and accumulates in the hepatopancreas, a key organ for detoxification (Das et al., 2023). Cd uptake was significantly greater under co-exposure conditions at PET + Cd (*p* < 0.05) in the current study. While this pattern is consistent with previous reports suggesting that MPs can adsorb trace metals and potentially facilitate their transport into aquatic organisms (Bhaumik and Chakraborty, 2024; Yang et al., 2022; Narwal et al., 2024). Although the study did not assess adsorption isotherms, surface binding, or desorption kinetics, the increased Cd accumulation should be considered a potential MP-associated vector effect rather than a confirmed adsorption mechanism.

Aquatic organisms often accumulate and inadvertently ingest toxicants, leading to physiological stress and organ dysfunction (Jahan et al., 2024b). Previous research indicates that MPs exposure in crustaceans significantly impairs growth and disrupts vital physiological processes (Lee et al., 2021). Our study found reduced growth and survival across all exposure groups compared to the control. MP accumulation has been associated with reduced feeding efficiency and altered energy balance in aquatic crustaceans (Zhang et al., 2023; Park et al., 2024; Xu et al., 2017). While the reduced growth matches similar findings, direct measurements of feeding rate or gut obstruction weren’t performed, so causal links should be interpreted cautiously. Survival varied among treatments, with PS exposure having the lowest survival rate, possibly due to PS’s physicochemical properties, such as hydrophobicity and interactions with contaminants (Huang et al., 2024). However, as surface adsorption characteristics were not experimentally evaluated in this study, this explanation remains speculative and is supported primarily by existing literature (Zhang et al., 2023; Park et al., 2024; Afsa et al., 2025). Moreover, PS may potentially cause gastrointestinal obstruction, reducing nutrient absorption and depleting energy reserves (Afsa et al., 2025). After 14 days, declines in SGR and survival reflected the combined toxicity of MP and Cd (Duan et al., 2021).

MPs detected in internal tissues, such as the hepatopancreas and muscle, may also be transported to the carapace and expelled during moulting (Pichardo-Casales et al., 2024). Moulting, a hormonally regulated physiological process essential for growth, can be disrupted by MP accumulation (Lee et al., 2021). The moulting process may serve as a defense mechanism, helping crustaceans eliminate MPs and mitigate their toxic effects (Pichardo-Casales et al., 2024). Crabs exposed to PET (200 μg/L) in the present experiment showed elevated Brix values, which indicate haemolymph sugar content. Brix levels typically rise before moulting and decline post-moult due to fluid intake (Battison, 2018). In addition, co-exposure to PET + Cd (200 μg/L + 62.5 μg/L) resulted in elevated brix values, possibly due to Cd’s interference with moulting hormone secretion (Luo et al., 2015). These findings align with current observations linking potential moulting difficulties and increased mortality to Cd toxicity, further supported by fluctuations in Brix values (Battison, 2018).

Haemolymph plays a crucial role in sustaining crustacean immune functions, enabling them to maintain health and resilience under diverse environmental conditions (Drolet et al., 2022). Its haematological profile is widely recognised as an indicator of nutritional status, stress response, and overall physiological health (Cherax et al., 2022). Moreover, haemolymph serves as a primary transport medium for toxicants such as heavy metals, facilitating their systemic distribution and toxicological impact (Capparelli et al., 2023). Among haemolymph biomarkers, ALT and AST are reliable indicators of tissue damage and are frequently used to assess hepatopancreatic function (Hamd et al., 2025; Banaee et al., 2025b). Numerous studies report that environmental stressors elevate ALT and AST levels in aquatic species (Cheng et al., 2017; Banaee et al., 2019). Specifically, Cd exposure significantly increases these enzyme activities, signaling hepatotoxicity and oxidative stress (Banaee et al., 2025a). This study observed elevated ALT and AST levels across all treatment groups compared to the control, indicating liver and pancreatic damage, as well as enzyme leakage. Oxidative stress caused by MPs and Cd could disrupt metabolism and elevate transaminase activity. Total cholesterol (TC) is essential for crustacean growth; however, high TC levels can hinder growth in mud crabs (*Scylla paramamosain*) (Liu et al., 2024; Wang et al., 2025). After 14 days, TG and TC increased in the PET, Cd, and co-exposure groups, indicating stress-related lipid buildup and disrupted metabolism. Additionally, higher glucose and TP levels further suggest physiological stress, with TP serving as a dependable biomarker, aligning with previous research (Battison, 2018; Qyli et al., 2020).

Histopathological analysis showed severe damage in the gills and hepatopancreas of exposed crabs. Gill lesions, such as epithelial lifting, lamellae fusion, clubbing, and rupture, could impair gas exchange and osmoregulation (Dias et al., 2024). Comparable abnormalities, such as epithelial collapse, hyperplasia, and lamellar hypertrophy, have been reported in *E. sinensis* and *Leptuca pugilator* following PS and Cu exposure (Xu et al., 2025). Cd exposure similarly induces gill surface wrinkling, lamellar disconnection, and hemocyte infiltration (Zhu et al., 2018). This study identified deformities and new abnormalities like lamellar rupture and clubbing. The hepatopancreas exhibited vacuolization, necrosis, and structural damage, which hinder digestion, absorption, and detoxification. Previous research also reported effects of MPs and metals, including hepatic tubular dilation and epithelial atrophy (Rainieri et al., 2018). Although PET-specific data are limited, PE studies suggest similar impacts (Guillén-Watson et al., 2023). Cd toxicity also causes hepatopancreatic injury, such as cell lysis and tissue detachment. Higher Cd accumulation in hepatopancreas supports its role in detoxification (Franchi et al., 2011). The differential tissue-specific toxicity of PS and PET-MPs in aquatic organisms can be attributed to their distinct physicochemical properties and the functional roles of target tissues (Sinha et al., 2026; Liu et al., 2026). Morphological and biochemical changes in female *S. spinigera* likely impaired their performance and metabolism, leading to slower growth and lower survival, especially under PS and PS + Cd conditions. PET may have affected hepatopancreas metabolism, but PS caused more growth inhibition and death. The higher mortality with PS is mainly due to residual styrene monomers, PAH additives, and PS surfaces acting as vectors for environmental contaminants, increasing toxicity beyond mechanical effects (Rochman et al., 2013a; Schweighuber et al., 2019; Besseling et al., 2014). Biochemical markers and tissue deformities were more severe under combined exposures (*p* < 0.05). Female *S. spinigera* crabs appear to be more sensitive to PS exposure in gill tissue, whereas PET possibly affected the hepatopancreas, reflecting differences in plastic properties and species responses. PS-MPs, with their hydrophobic aromatic surface, mainly accumulate in gill tissues, blocking lamellae and causing oxidative stress, as shown in previous studies (Umamaheswari et al., 2021; Kaloyianni et al., 2021; Sun et al., 2024). In contrast, PET-MPs, which contain ester linkages that can leach oligomers and additives in aquatic environments, are more readily taken up by endocytosis in hepatopancreatic cells (Yoo et al., 2024; Lu et al., 2025). This process could pose risks, including immune system toxicity, effects on energy metabolism, lipid peroxidation, and disruption of antioxidant enzyme activity (Yoo et al., 2024; Lu et al., 2025). Additionally, the fragmentation potential of these polymers improves their bioavailability and cell entry, enabling them to breach epithelial barriers and induce mitochondrial dysfunction and apoptosis in sensitive tissues (Sultan et al., 2024; Rochman et al., 2013b).

PCA revealed that exposure to PS, PET, and Cd, either alone or in combination, induced physiological and metabolic stress in *S. spinigera*. Controls showed better survival and growth, while PS and Cd increased liver enzymes (AST, ALT), indicating liver damage. PET and PET + Cd disrupted metabolic markers, suggesting energy issues. MPs, especially with Cd, affect freshwater crabs’ behaviour, physiology, haemato-biochemical profiles, and organs. Their combined toxicity poses ecological risks and links contamination to crustacean decline, underscoring the importance of mitigation and regulation.

## 5 Conclusion

Exposure to MPs (PS and PET) and Cd, alone or combined, markedly reduced growth and survival in female *S. spinigera*. Bioaccumulation occurred in the gills and hepatopancreas, with PET favouring hepatopancreatic accumulation and PS showing greater retention in gills. Toxicant exposure elevated hematobiochemical markers (ALT, AST, TC, TG, TP, Glucose, and Brix) and reduced survival. Based on bioaccumulation and histomorphological changes, PS may cause severe gill damage, while PET affected the hepatopancreas. These physiological effects, including reduced growth, survival, and organ damage, may negatively impact reproduction and recruitment, potentially leading to population decline. Further research is required to clarify the mechanisms of MP and HM invasion in *S. spinigera* and the factors influencing their absorption into internal tissues, especially within larger, ecologically realistic systems. This study did not control water evaporation, simulate trophic bioaccumulation, or measure biomarkers like ecdysone and vitellogenin. Crabs couldn’t avoid toxins, possibly causing stress-related behavioral and physiological changes. Future work should examine uptake pathways, tissue-specific accumulation, adsorption amount of trace metals via MPs, and impacts under full-scale ecosystem simulations.

## Data Availability

The original contributions presented in the study are included in the article/Supplementary Material, further inquiries can be directed to the corresponding authors.
