# Supplementary material for: Toxicological impacts of environmentally equivalent microplastics and cadmium co-exposure in tropical freshwater crab Sartoriana spinigera
Source: Front Toxicol. 2026 May 28;8:1804866. doi: 10.3389/ftox.2026.1804866 (PMC13252950; doi:10.3389/ftox.2026.1804866)
Supplement: Supplementary file 1 [file Supplementaryfile1.docx]

**Supplementary materials**

**Table S1** An overview of the experimental design outlining the allocation of freshwater crab *S. spinigera* to each treatment group for assessing the effects of PS, PET, and Cd exposure.

| Treatment | PS (µg/L) | PET (µg/L) | CdCl_2_ (µg/L) | Replications of each treatment | Number of crabs per treatment | Total number of crabs |
| --- | --- | --- | --- | --- | --- | --- |
| Control | 0 | 0 | 0 | 3 | 15 | 90 |
| PS | 200 | 0 | 0 |  |  |  |
| PET | 0 | 200 | 0 |  |  |  |
| Cd | 0 | 0 | 62.5 |  |  |  |
| PS + Cd | 200 | 0 | 62.5 |  |  |  |
| PET + Cd | 0 | 200 | 62.5 |  |  |  |

**Table S2** Physicochemical parameters of water in different treatment groups during the exposure period (values are presented as mean ± SEM).

| Treatments | Period | Temperature (°C) | Dissolved Oxygen (%) | Dissolved Oxygen (ppt) | Electric Conductivity (µScm^−1^) | Total Dissolved Solids (mgL^-1^) | pH | Salinity | NH_3_  (mgL^-1^) |
| --- | --- | --- | --- | --- | --- | --- | --- | --- | --- |
| Control | 7 days | 28.60±0.25 | 87.84±1.50 | 6.75±0.14 | 159.90±1.10 | 73.96±0.44 | 7.35±0.04 | 0.07±0.00 | 0.08±0.02 |
| PS |  | 28.66±0.14 | 89.13±1.61 | 6.76±0.16 | 159.74±1.40 | 73.52±0.48 | 7.35±0.05 | 0.07±0.00 | 0.06±0.01 |
| PET |  | 29.00±0.07 | 88.04±1.21 | 6.64±0.13 | 160.11±1.17 | 73.42±0.45 | 7.30±0.03 | 0.07±0.00 | 0.07±0.02 |
| Cd |  | 28.70±0.11 | 88.51±1.03 | 6.69±0.12 | 161.06±1.40 | 74.01±0.38 | 7.24±0.01 | 0.07±0.00 | 0.06±0.01 |
| PS + Cd |  | 28.82±0.19 | 88.79±1.18 | 6.82±0.10 | 159.38±0.93 | 73.94±0.38 | 7.29±0.02 | 0.07±0.00 | 0.05±0.02 |
| PET + Cd |  | 28.63±0.05 | 88.47±1.00 | 6.82±0.08 | 159.46±0.54 | 73.91±0.38 | 7.24±0.01 | 0.07±0.00 | 0.07±0.02 |
| Mean |  | 28.74±0.06 | 88.46±0.50 | 6.75±0.05 | 159.94±0.44 | 73.79±0.17 | 7.30±0.01 | 0.07±0.00 | 0.06±0.01 |
| Control | 14 days | 28.81±0.16 | 94.23±0.30 | 7.26±0.02 | 159.90±1.29 | 74.48±0.44 | 6.74±0.07 | 0.07±0.00 | 0.04±0.01 |
| PS |  | 28.93±0.16 | 92.63±0.34 | 7.13±0.04 | 159.40±1.44 | 74.07±0.49 | 6.73±0.07 | 0.07±0.00 | 0.05±0.01 |
| PET |  | 29.24±0.16 | 93.18±0.43 | 7.15±0.02 | 156.71±0.87 | 72.14±0.21 | 6.73±0.07 | 0.07±0.00 | 0.03±0.01 |
| Cd |  | 29.33±0.17 | 91.74±0.42 | 7.01±0.02 | 159.19±1.11 | 73.54±0.38 | 6.73±0.07 | 0.07±0.00 | 0.04±0.01 |
| PS + Cd |  | 29.06±0.19 | 92.03±0.58 | 7.09±0.03 | 159.03±1.49 | 73.86±0.53 | 6.74±0.07 | 0.07±0.00 | 0.02±0.00 |
| PET + Cd |  | 29.16±0.15 | 92.04±0.64 | 7.06±0.04 | 161.26±1.06 | 74.63±0.38 | 6.73±0.07 | 0.07±0.00 | 0.02±0.00 |
| Mean |  | 29.09±0.07 | 92.64±0.22 | 7.12±0.02 | 159.25±0.51 | 73.79±0.20 | 6.73±0.03 | 0.07±0.00 | 0.03±0.00 |

**Table S3** Behavioral alterations observed in Female S. spinigera under stress conditions, with descriptions based on the literature.

| **Behavioral Changes** | **Description (Based on Literature)** | **Reference** |
| --- | --- | --- |
| Aggregation | Association and collective grouping of crabs as a form of social or protective behavior. | [1,2] |
| Aggression | Display of hostile or aggressive behaviour among individuals. | [1] |
| Attraction towards food | Feeding activity directed toward available food sources. | [1] |
| Fecal matter | Excretion of waste material resulting from digestive processes. | [1] |
| Froth-releasing activity | Emission of white froth or bubbles from the mouthparts as part of respiratory and cleaning activity. | [2] |
| Hiding tendency | Retreat or concealment in corners behind 4 aquarium air-bubble stone. | Current study |
| Locomotion | Movement or displacement of crabs within their environment. | [3] |
| Mouth part activity | Movement of mouthparts related to feeding and ventilation functions. | [2,3] |
| Movement of chelate legs | The chelate legs around the mouthparts perform cleaning or grooming movements. | [3] |
| Riding tendency | Climbing behavior where crabs move over each other or objects. | [2] |

**Table S4** Mean ± standard deviation of microplastic recovery rates obtained using the sample digestion protocol (10% KOH, incubated at 40°C for 5 days) applied to spiked samples (n = 3).

| **Polymers (shape)** | **Replicates** | **Tissue (1 g)** | **Particles Added** | **Particles Recovered** | **Recovery (%)** |
| --- | --- | --- | --- | --- | --- |
| PS (irregular fragments) | 1 | Gill | 50 | 48 | 96.00% |
|  | 2 |  |  | 48 | 96.00% |
|  | 3 |  |  | 47 | 94.00% |
|  | 1 | Hepatopancreas |  | 46 | 92.00% |
|  | 2 |  |  | 46 | 92.00% |
|  | 3 |  |  | 47 | 94.00% |
| PET (irregular fragments) | 1 | Gill |  | 49 | 98.00% |
|  | 2 |  |  | 48 | 96.00% |
|  | 3 |  |  | 49 | 98.00% |
|  | 1 | Hepatopancreas |  | 47 | 94.00% |
|  | 2 |  |  | 46 | 92.00% |
|  | 3 |  |  | 45 | 90.00% |
| Overall Recovery (%); Mean | | | | | 94.33% |
| SD | | | | | 2.53% |

**Table S5** Detailed statistical outputs for all analyses conducted in the experiment (test statistics, degrees of freedom, and exact *p*-values results).

| **Remarks** |  | **Sum of Squares** | **Degrees of freedom (*df*)** | **Mean Square** | **F** | **Sig. (*p*-value)** |
| --- | --- | --- | --- | --- | --- | --- |
| **Growth Parameters** | | | | | | |
| Initial Weight (g) | Between Groups | 0.025 | 5 | 0.005 | 0.004 | 1.000 |
|  | Within Groups | 67.956 | 48 | 1.416 |  |  |
| Final Weight (g) | Between Groups | 5.966 | 5 | 1.193 | 0.732 | 0.604 |
|  | Within Groups | 61.932 | 38 | 1.630 |  |  |
| Specific Growth Rate (SGR) % | Between Groups | 1.101 | 5 | 0.220 | 14.104 | 0.000 |
|  | Within Groups | 0.593 | 38 | 0.016 |  |  |
| Hepatopancreas Weight (g) | Between Groups | 0.056 | 5 | 0.011 | 1.441 | 0.232 |
|  | Within Groups | 0.295 | 38 | 0.008 |  |  |
| Hepatopancreatic Index | Between Groups | 2.343 | 5 | 0.469 | 1.649 | 0.171 |
|  | Within Groups | 10.797 | 38 | 0.284 |  |  |
| Survival Rate (%) | Between Groups | 9533.333 | 5 | 1906.667 | 21.450 | 0.000 |
|  | Within Groups | 1066.667 | 12 | 88.889 |  |  |
| **MPs and Trace Metals Bioaccumulation** | | | | | | |
| MP in Gills (items/g ww) | Between Groups | 1322.000 | 3 | 440.667 | 20.496 | 0.000 |
|  | Within Groups | 172.000 | 8 | 21.500 |  |  |
| MP in Hepatopancreas (items/g ww) | Between Groups | 828.917 | 3 | 276.306 | 35.652 | 0.000 |
|  | Within Groups | 62.000 | 8 | 7.750 |  |  |
| Cd in whole sample (µg/g dry weight) | Between Groups | 0.024 | 2 | 0.012 | 202.482 | 0.000 |
|  | Within Groups | 0.000 | 6 | 0.000 |  |  |
| **Hemato-Biochemical Parameters** | | | | | | |
| ALT (U/L) | Between Groups | 37.265 | 5 | 7.453 | 22.128 | 0.000 |
|  | Within Groups | 4.042 | 12 | 0.337 |  |  |
| AST (U/L) | Between Groups | 561.444 | 5 | 112.289 | 161.696 | 0.000 |
|  | Within Groups | 8.333 | 12 | 0.694 |  |  |
| Total Cholesterol (mg/dl) | Between Groups | 25.279 | 5 | 5.056 | 122.068 | 0.000 |
|  | Within Groups | 0.497 | 12 | 0.041 |  |  |
| Triglycerides (mmol/L) | Between Groups | 0.332 | 5 | 0.066 | 33.407 | 0.000 |
|  | Within Groups | 0.024 | 12 | 0.002 |  |  |
| Total Protein (g/dl) | Between Groups | 9.538 | 5 | 1.908 | 7.804 | 0.002 |
|  | Within Groups | 2.933 | 12 | 0.244 |  |  |
| Glucose (mg/dl) | Between Groups | 5038.020 | 5 | 1007.604 | 16.040 | 0.000 |
|  | Within Groups | 753.840 | 12 | 62.820 |  |  |
| Brix (%) | Between Groups | 200.191 | 5 | 40.038 | 4.653 | 0.014 |
|  | Within Groups | 103.247 | 12 | 8.604 |  |  |

**Table S6** The proportion of behavioural abnormalities in *S. spinigera* was exposed to individual and combined exposure of MPs and Cd for 14 days.

| Treatments | Day | Behavioral changes (%) | | | | | | | | | |
| --- | --- | --- | --- | --- | --- | --- | --- | --- | --- | --- | --- |
|  |  | Aggregation | Aggression | Attraction towards food | Fecal matter | Froth-releasing activity | Hiding tendency | Locomotion | Mouth part activity | Movement of chelate legs | Riding tendency |
| Control | 7 | 43.93 | 24.88 | 48.89 | 44.37 | 50.83 | 31.19 | 51.43 | 31.75 | 32.78 | 53.77 |
| PS |  | 46.19 | 34.84 | 34.05 | 33.93 | 20.12 | 47.50 | 30.75 | 49.40 | 29.33 | 23.45 |
| PET |  | 44.29 | 20.60 | 45.67 | 47.86 | 33.57 | 52.14 | 37.90 | 28.97 | 36.35 | 19.92 |
| Cd |  | 50.36 | 40.95 | 24.56 | 42.34 | 23.93 | 63.53 | 46.43 | 54.92 | 38.25 | 45.95 |
| PS+Cd |  | 39.92 | 40.32 | 43.77 | 43.29 | 26.59 | 62.30 | 21.59 | 31.67 | 23.06 | 41.03 |
| PET+Cd |  | 48.93 | 31.31 | 43.41 | 42.42 | 35.36 | 68.10 | 29.21 | 36.90 | 43.61 | 41.63 |
| Control | 14 | 53.29 | 55.32 | 53.41 | 53.29 | 36.55 | 39.05 | 67.30 | 40.91 | 50.95 | 48.25 |
| PS |  | 26.59 | 7.62 | 26.27 | 34.48 | 12.58 | 65.04 | 19.09 | 36.83 | 18.89 | 2.86 |
| PET |  | 15.12 | 23.17 | 45.75 | 47.74 | 10.99 | 68.02 | 33.85 | 21.87 | 26.47 | 30.91 |
| Cd |  | 46.31 | 36.47 | 13.57 | 48.61 | 23.77 | 43.61 | 39.33 | 47.46 | 38.61 | 36.31 |
| PS+Cd |  | 36.35 | 17.30 | 25.79 | 36.51 | 19.44 | 63.77 | 14.64 | 24.68 | 22.14 | 26.71 |
| PET+Cd |  | 36.67 | 19.05 | 30.79 | 34.21 | 18.93 | 59.37 | 17.46 | 28.77 | 28.81 | 26.67 |

Behavioural changes were recorded in minutes and then calculated as percentages in the current analysis. The percentage was calculated from the frequency observed on 7 days and 14 days.

**Table S7** Percentage of histomorphological changes in female *S. spinigera* exposed to MPs and Cd for 14 days.

| Organs | Histological Abnormalities | Treatments | | | | | |
| --- | --- | --- | --- | --- | --- | --- | --- |
|  |  | Control | PS | PET | Cd | PS+Cd | PET+Cd |
| Gill | Clubbing | 0.00 | 54.44 | 6.67 | 54.44 | 35.56 | 63.33 |
|  | Connection of lamellae | 2.22 | 32.22 | 12.22 | 58.89 | 41.11 | 53.33 |
|  | Necrosis | 0.00 | 52.22 | 40.00 | 45.56 | 53.33 | 32.22 |
|  | Hyperplasia | 0.00 | 51.11 | 56.67 | 14.44 | 27.78 | 33.33 |
|  | Ruptured lamellae | 1.11 | 20.00 | 52.22 | 13.33 | 53.33 | 42.22 |
|  | Detached lamellae | 0.00 | 21.11 | 10.00 | 5.56 | 68.89 | 6.67 |
|  | Swollen lamellae | 2.22 | 34.44 | 7.78 | 22.22 | 58.89 | 31.11 |
| Hepatopancreas | Vacuoles | 1.11 | 32.22 | 71.11 | 67.78 | 47.78 | 65.56 |
|  | Dilation of hepatic tubular lumen | 1.11 | 26.67 | 51.11 | 41.11 | 54.44 | 45.56 |
|  | Cell lysis | 0.00 | 3.33 | 31.11 | 57.78 | 28.89 | 58.89 |
|  | Hepatic digestion | 0.00 | 14.44 | 52.22 | 27.78 | 43.33 | 62.22 |

< 5% has been referred to as absent (–), 5 – 25% as weak (+), > 25 – 50% as moderate (++), and > 50 % as severe (+++) in the current study.

| 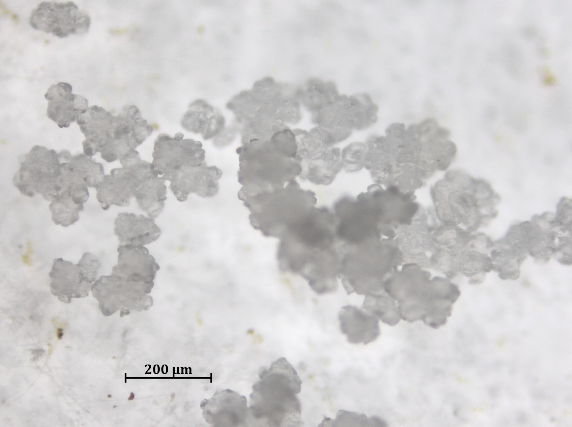 | 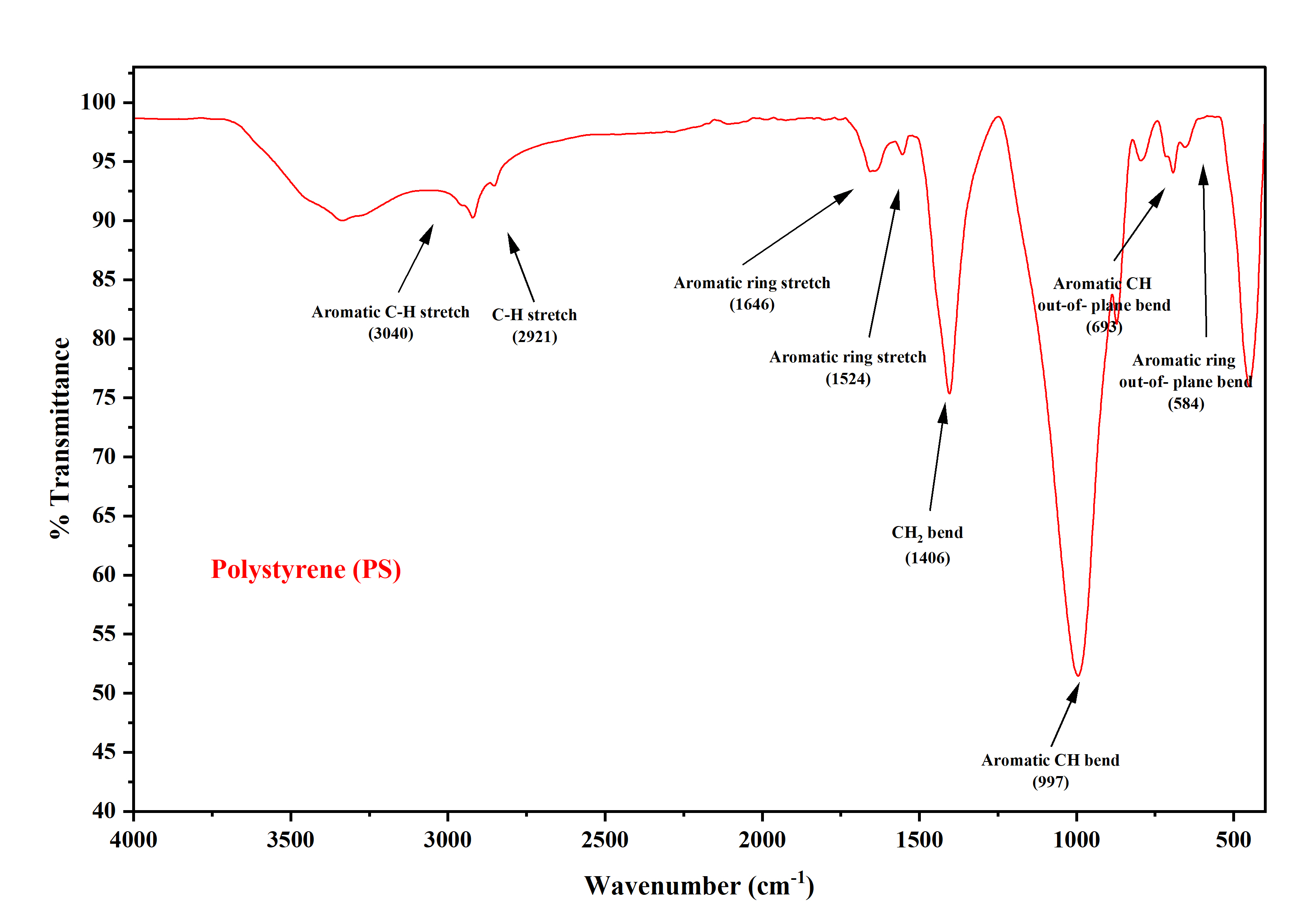 |
| --- | --- |
| **(A) Polystyrene (PS)** | |
| 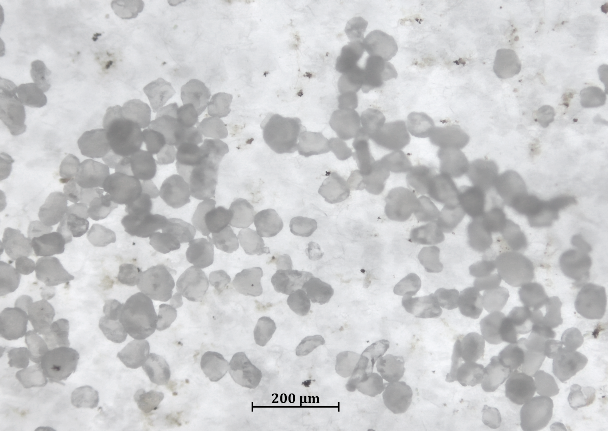 | 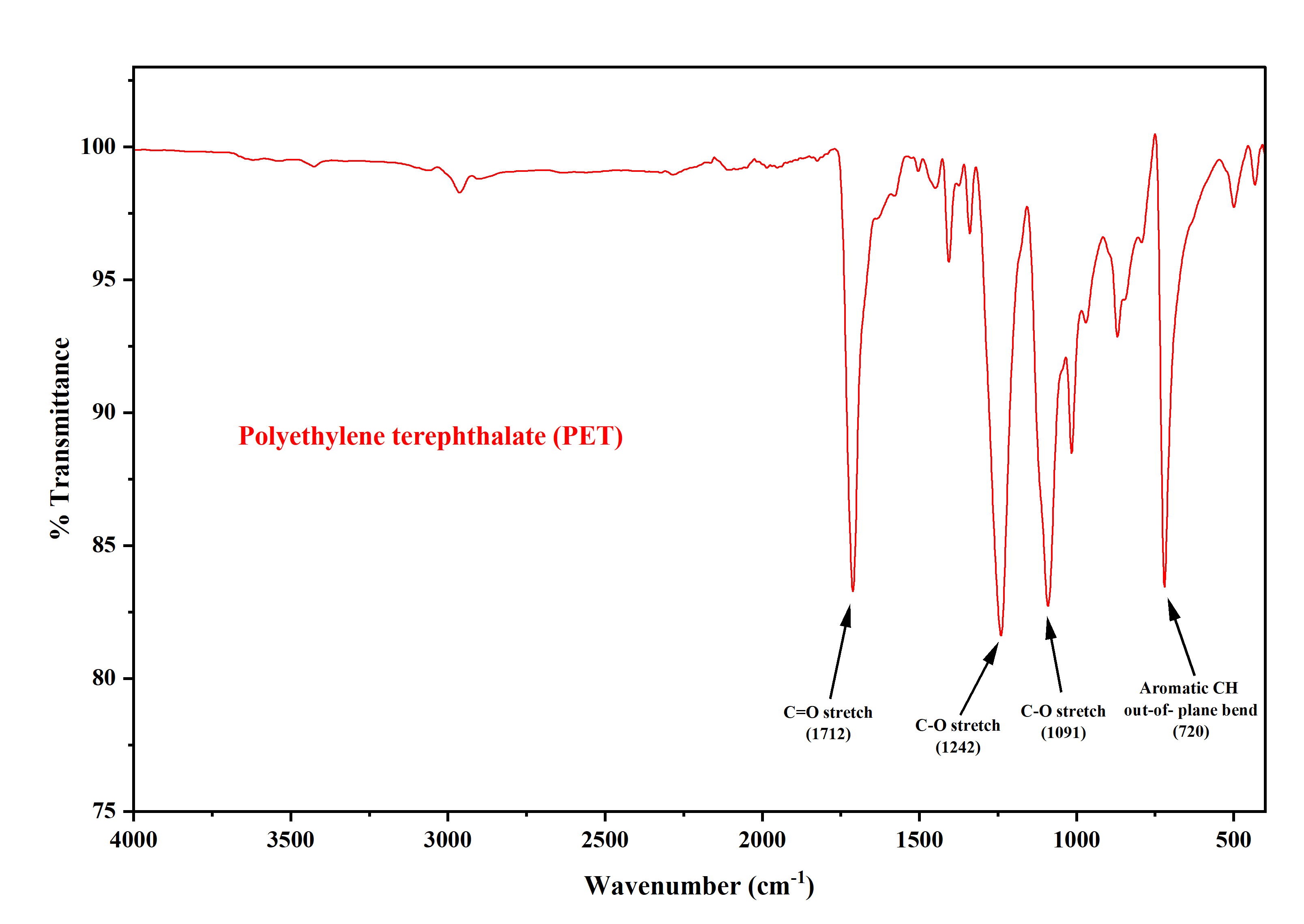 |
| **(B) Polyethylene terephthalate (PET)** | |

**Figure S1** FTIR spectra of procured microplastic polymer materials. FTIR spectra of the two procured polymer materials, (A) polystyrene (PS) and (B) polyethylene terephthalate (PET), obtained via Fourier-transform infrared (FTIR) spectroscopy. Key characteristic absorption bands confirm the identity of each polymer with reference spectra [4]; PS is identified by its aromatic C–H stretching and aromatic bending vibrations, while PET is identified by its characteristic C=O, and C–O stretching, which validate the composition of the commercially procured materials as labelled by the supplier.

|  | PS | PS + Cd | PET | PET + Cd |
| --- | --- | --- | --- | --- |
| Gill | 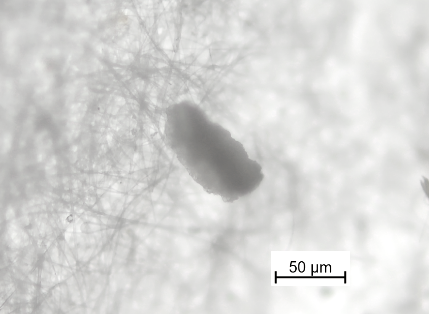  **A** | 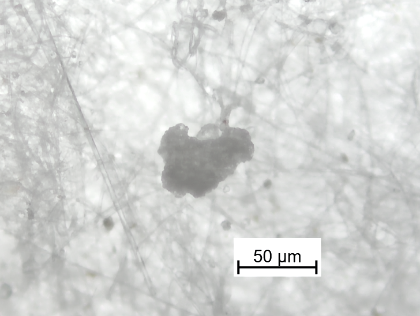  **B** | 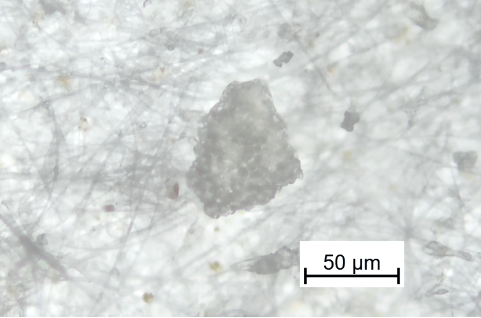  **C** | 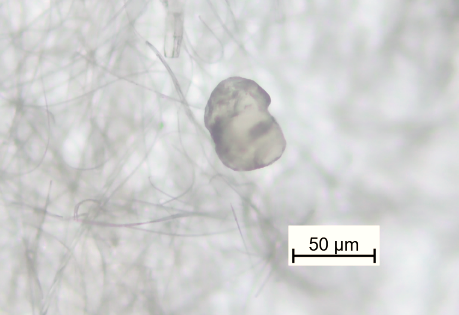  **D** |
| Hepatopancreas | 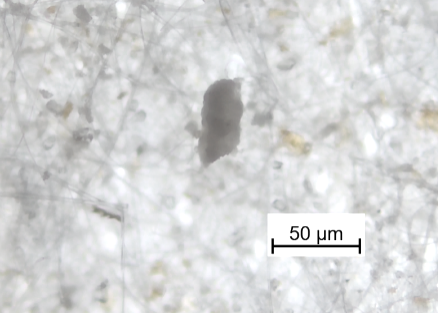  **E** | 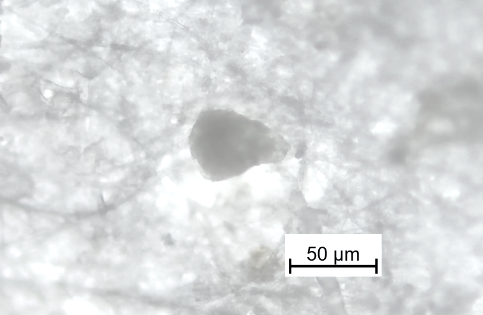  **F** | 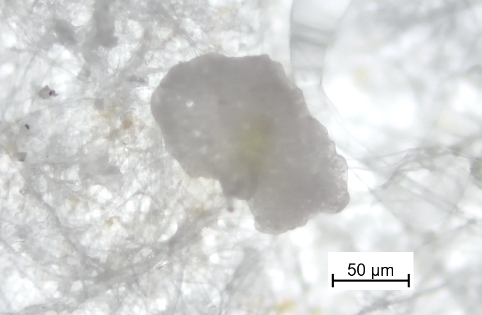  **G** | 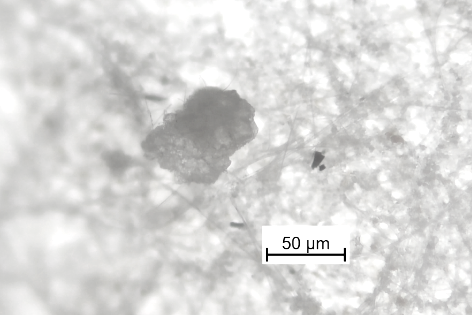  **H** |
| MPs employed in the trial | 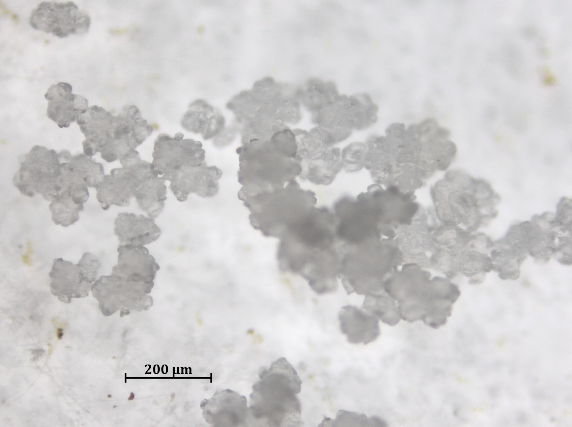  **I** | | 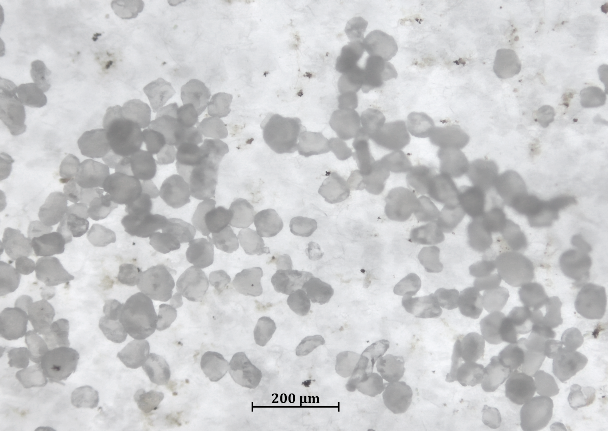  **J** | |

**Figure S2**. MP particles observed under a stereomicroscope, including those applied in the experiment and those bioaccumulated by the organisms**.** MPs found in gills: (A) PS, (B) PS + Cd, (C) PET, (D) PET + Cd; MPs found in hepatopancreas: (E) PE, (F) PS + Cd, (G) PET, (H) PET + Cd; MPs applied in trial: (I) PS, (J) PET. Each image includes scale bars. Magnifications are set at 4x and 10x, with scale bars measuring 200 µm and 50 µm, respectively.

**References**

1. Rosaria, J.C.; Martin, E.R. Behavioral Changes in Freshwater Crab, *Barytelphusa Cunicularis* after Exposure to Low Frequency Electromagnetic Fields. *World J. Fish Mar. Sci.* **2010**, *2*, 487–494.

2. Deyashi, M.; Misra, K.K.; Chakraborty, S.B. Evaluation of the Acute Toxicity of Mahua Oil Cake Aqueous Extract and Its Effect on the Behavioral Responses of the Freshwater Grapsid Crab, *Varuna Litterata* (Fabricius, 1798). *Environ. Sci. Pollut. Res.* **2019**, *26*, 15631–15640, doi:10.1007/s11356-019-04930-8.

3. Mcgaw, I.J.; Reiber, C.L.; Guadagnoli, J.A. Behavioral Physiology of Four Crab Species in Low Salinity. *Biol. Bull.* **1999**, *196*, 163–176, doi:10.2307/1542562.

4. Jung, M.R.; Horgen, F.D.; Orski, S. V.; Rodriguez C., V.; Beers, K.L.; Balazs, G.H.; Jones, T.T.; Work, T.M.; Brignac, K.C.; Royer, S.-J.; et al. Validation of ATR FT-IR to Identify Polymers of Plastic Marine Debris, Including Those Ingested by Marine Organisms. *Mar. Pollut. Bull.* **2018**, *127*, 704–716, doi:10.1016/j.marpolbul.2017.12.061.
